# ID 29 - Extrapolação das Curvas de Sobrevida na Oncologia: revisão dos modelos econômicos da Conitec

**DOI:** 10.5327/2237-9622.2025.v34s1.29

**Published:** 2025-11-25

**Authors:** Stéfani Sousa Borges, Mateus Rodrigues Cerqueira, Gustavo Saraiva Frio, Bruna Bento dos Santos, Ivan Ricardo Zimmermann

**Keywords:** avaliação econômica em saúde, análise de sobrevida, modelagem, tratamento oncológico

## Abstract

**Introdução:**

A modelagem econômica na oncologia frequentemente enfrenta desafios na estimativa de benefícios clínicos como a sobrevida global (SG) e a sobrevida livre de progressão (SLP), especialmente quando as evidências vêm de ensaios clínicos de curta duração. Nesses casos, a estratégia baseia-se na extrapolação das curvas de sobrevida para além do tempo de observação. A escolha do método de modelagem adequado é uma tarefa complexa, que exige rigor para garantir estimativas precisas e confiáveis. No contexto da Avaliação de Tecnologias em Saúde (ATS) realizada pela Comissão Nacional de Incorporação de Tecnologias no Sistema Único de Saúde (Conitec), essas estimativas são essenciais para recomendações informadas sobre a incorporação de tecnologias no tratamento oncológico. Este estudo visa analisar os métodos utilizados na ATS de medicamentos oncológicos e avaliar padrões e práticas de modelagem.

**Método:**

Estudo descritivo das avaliações econômicas dos relatórios de recomendação sobre medicamentos oncológicos da Conitec, publicados entre janeiro de 2019 e dezembro de 2023. Três revisores independentes selecionaram os relatórios e extraíram os dados de interesse usando um formulário próprio contendo os parâmetros: desfechos elencados, curvas de distribuição adotadas na extrapolação de sobrevida, métodos de validação e horizonte temporal.

**Resultados:**

Foram identificados 30 relatórios de recomendação da Conitec, avaliando 32 tratamentos oncológicos para 45 diferentes indicações. Os modelos econômicos mais usados foram a Análise de Sobrevida Particionada (PartSA) (58%) e Markov (33%). A estrutura de estados transicionais SLP, progressão e morte foi reportada em 73% dos modelos PartSA e 67% dos modelos de Markov. Na PartSA, a distribuição Weibull foi adotada em 50% das extrapolações de SG, enquanto Exponencial (35%) e Log-normal (15%) em extrapolações de SLP. Nos modelos de Markov a distribuição Weibull (84%) foi mais frequente, seguida de Log normal (54%) e Exponencial (8%). Métodos de validação foram reportados em 69% dos modelos de Markov e 100% das PartSA. Os horizontes de extrapolação mais comuns foram 10 anos (75% dos modelos), 15 anos (31%) e 30 anos (31%).

**Conclusão:**

A utilização dos modelos de Markov e PartSA é justificada por sua capacidade de modelar a progressão do câncer ao longo do tempo. No entanto, o Markov pode não capturar todas as nuances dos tratamentos complexos e o PartSA exige dados de alta qualidade para extrapolações de longo prazo. Foi observada preferência pela distribuição Weibull para extrapolações de SG e Exponencial para SLP. Apesar das limitações, esses modelos são amplamente aceitos devido à sua capacidade de representar satisfatoriamente a progressão do câncer e sublinha a importância de considerar a complexidade dos tratamentos e a influência de novas tecnologias na avaliação econômica, destacando que limiares de custo-efetividade não são determinantes absolutos nas recomendações de incorporação.

